# Comparative mRNA Expression Profiles of Riboflavin Biosynthesis Genes in Lactobacilli Isolated from Human Feces and Fermented Bamboo Shoots

**DOI:** 10.3389/fmicb.2017.00427

**Published:** 2017-03-17

**Authors:** Kiran Thakur, Sudhir K. Tomar, Zhao-Jun Wei

**Affiliations:** ^1^Dairy Microbiology Division, Indian Council of Agricultural Research – National Dairy Research InstituteKarnal, India; ^2^School of Food Science and Engineering, Hefei University of TechnologyHefei, China

**Keywords:** riboflavin, lactobacilli, fermented bamboo shoots, milk, whey, mRNA, roseoflavin

## Abstract

With the aim to bioprospect potent riboflavin producing lactobacilli, the present study was carried out to evaluate the relative mRNA expression of riboflavin biosynthesis genes namely *Rib* 1, *Rib* 2, *Rib* 3, and *Rib* 4 from potent riboflavin producers obtained from our previous studies. All the four genes were successfully cloned and sequenced for further analysis by *in silico* procedures. As studied by non-denaturing Polyacrylamide gel electrophoresis, no difference in size of all the four genes among those of various lactobacilli was observed. The relative fold increase in mRNA expression in *Rib* 1, *Rib* 2, *Rib* 3, and *Rib* 4 genes has been observed to be 10-, 1-, 0.7-, and 8.5-fold, respectively. Due to increase in relative mRNA expression for all the *Rib* genes as well as phenotypic production attribute, KTLF1 strain was used further for expression studies in milk and whey. The fold increase in mRNA expression for all the four *Rib* genes was higher at 12 and 18 h in milk and whey respectively. After exposure to roseoflavin, resistant variant of KTLF1 showed considerable increase in expression of all the targets genes. This is the first ever study to compare the mRNA expression of riboflavin biosynthesis pathway genes in lactobacilli and it also under lines the effect of media and harvesting time which significantly affect the expression of *rib* genes. The use of roseoflavin-resistant strains capable of synthesizing riboflavin in milk and whey paves a way for an exciting and economically viable biotechnological approach to develop novel riboflavin bio-enriched functional foods.

## Introduction

In the recent years, many researchers have shown burgeoning interest in riboflavin which has now regarded as an essential component of cellular biochemistry (Thakur et al., 2016a). Several bacteria have the trait to synthesize riboflavin and its pathway has been studied in bacteria whereas humans lack its biosynthesis ability (Perkins and Pero, 2002). The microorganisms harbor the genetic structure to synthesize B vitamins particularly riboflavin to obtain bio-enriched food (Capozzi et al., 2011). Due to adaptability to fermentation processes, lactic acid bacteria (LAB) act an ideal candidates for *in situ* riboflavin production in food (Arena et al., 2014). Though, ability for riboflavin biosynthesis is strain specific (Capozzi et al., 2012). An alternative RNA structure involving the RFN element serves a model for regulation of riboflavin biosynthesis (Gelfand et al., 1999; Vitreschak et al., 2002). Riboflavin metabolism and transport genes are regulated at transcription attenuation and translation initiation level in Gram-positive bacteria and Gram-negative bacteria respectively (Vitreschak et al., 2002). Four genes (*rib1, rib2, rib3*, and *rib4*) are required for biosynthesis of riboflavin from guanosine triphosphate (GTP) and ribulose-5- phosphate (Perkins et al., 1999). According to these authors, these genes are located in an operon and their order differs from that of enzymatic reactions (Richter et al., 1992, 1993, 1997). There are mainly two promoters responsible for transcription of riboflavin genes where all the four genes are controlled are primarily controlled by the *rib*P1 promoter (Perkins et al., 1999). The *rib*3 and *rib*4, are regulated from a second promoter (*rib*P2) and regulatory region RFN (Perkins et al., 1999).

There are number of reports where overexpression in riboflavin production was observed after exposure to range of roseoflavin (a chemical analog to riboflavin) (Burgess et al., 2004, 2006; del Valle et al., 2014). In these studies, riboflavin overproduction directly correlated with the spontaneous roseoflavin resistant strains (Burgess et al., 2006; Capozzi et al., 2011). The tolerance to the toxic roseoflavin signifies the mutations in the regulatory region of the rib operon which ultimately give rise to riboflavin over producing phenotype. Lately, *in situ* bacterial overproduction of the B group vitamins, including riboflavin is of significant interest (Burgess et al., 2009; Capozzi et al., 2012). In particular for riboflavin, promising results have been reported for the production of yogurt (Burgess et al., 2006) or pasta and bread (Capozzi et al., 2011; Arena et al., 2014) and Soymilk (del Valle et al., 2014). Many researchers (Jayashree et al., 2011; Guru and Viswanathan, 2013; del Valle et al., 2014; Thakur and Tomar, 2015a; Thakur et al., 2016c) have studied the riboflavin production in LAB in MRS, Riboflavin free media, milk and whey but no one has ever reported the expression levels of riboflavin biosynthesis genes. The Lactobacilli used for present study were previously isolated and identified from various niches (human feces, fermented bamboo shoots, and curd) (Thakur and Tomar, 2015a; Thakur et al., 2015a, 2016c). Among them Lactobacilli isolated from fermented bamboo shoots (Manipur, India) have shown highest riboflavin producing properties as well as displayed probiotic and appreciable techno-functional properties (Thakur et al., 2015a). In the continuance of our previous reports, the present study reveals the first ever profile of mRNA expression of four *Rib* genes (molecular determinants for riboflavin biosynthesis which form a complete functional *rib* operon) in four different media by harvesting the test isolates at different intervals of time. There are few reports where the regulatory mechanism of riboflavin biosynthesis has been studied in roseoflavin resistant variants in LAB. However, there exists *per se* no such report for *Lactobacillus* species till date.

## Materials and Methods

### Bacterial Strains and Growth Conditions

The *Lactobacillus* strains (**Table 1**) used in this work were confirmed for riboflavin production by an array of analytical methods viz. Polymerase chain reaction (PCR) based method (presence of riboflavin biosynthesis genes), Spectrophotometric method, Microbiological assay method, and High Performance Liquid Chromatography in our previous studies (Thakur and Tomar, 2015a; Thakur et al., 2016b). All the strains stored previously at -80°C in MRS supplemented with glycerol (20% v/v) were routinely cultured on de Man-Rogosa -Sharp (MRS) medium (Sigma- Aldrich, St. Louis, MO, USA) for this study.

**Table 1:** Isolates used in this study.

Sr. No.	Genus	Species	Given name	Source
1	*Lactobacillus*	*fermentum*	KTLF1	Our previous studies
2	*Lactobacillus*	*fermentum*	KTLF3	
3	*Lactobacillus*	*plantarum*	KTP13	
4	*Lactobacillus*	*mucosae*	KT2	
5 (Standard)	*Lactobacillus*	*fermentum*	MTCC8711	MTCC, Chandigarh, India


### Cloning, Transformation, and Sequencing

Purified PCR products (HiPuraA^TM^ purification kit, Himedia, India) were used for cloning of all the four genes. The cloning vector used in this study was _P_TZ57R/T clone vector amp (InstClone PCR cloning kit, Stratagene, USA). The clones were transformed into competent cells of *Escherichia coli* (*E. coli*) (XL1 blue). The successful clones were picked from Luria broth+ ampicillin plates and amplified for target genes by colony PCR method followed by plasmid isolation. The positive clones were identified by PCR analysis of plasmid DNA by using primers used in our previous study (Thakur et al., 2016b). The nucleotide sequencing was performed by sequencing services provided by Xcelris Labs, Ltd, Ahmedabad, India. The chromatograms of sequences obtained were analyzed and converted to Fasta using Bio-Edit Software. Nucleotide sequence similarity searches were performed for the obtained sequences by matching with previously published complete genome of *Lactobacillus* species of interest.

### Size Variation in Rib Genes by Polyacrylamide Gel Electrophoresis (PAGE)

Non-denaturing PAGE was used to detect the difference is size of all the four *Rib* genes amplified in different lactobacilli. Silver staining was used to view the band pattern in the PAGE after the final gel run.

### Growth in MRS, RAM, Milk and Whey Based Media

The test isolates were washed thrice with saline solution (0.85% m/v NaCl), resuspended in this solution and used to inoculate at 2% (v/v) riboflavin-free culture medium (Riboflavin Assay Medium, Difco, Becton, Dickinson, and Co., Sparks, MD, USA), reconstituted skim milk and whey based medium and then incubated without agitation at 37°C for 18 h. The optical density of selected isolates was observed before harvesting them for RNA isolation. The log count/ml was checked at lag, log, and stationary phases of growth.

### Designing of Primers

The primers (**Table 2**) were designed by aligning sequences of riboflavin operon using CLUSTALW program. The *Lactobacillus fermentum* IFO3956 strain was considered for primer selection: GenBank accession number NC_010610. The house keeping genes for normalizing real time reaction were synthesized for *REC* gene essential for the repair and maintenance of DNA and *TUF* gene encoding elongation factor from the database of genome in NCBI.

**Table 2:** Real-time (RT-PCR) primers designed for this study.

Gene name	Primer sequence	Melting temperature	Product size	Reference
Rib1	F′ GGCAGTCATTCGGGGTGCAACCG R′ CTTAAAGCCAGCGCGATCCATAGCTTGTTC	62 63	157 bp	Present study
Rib2	F′ CCGGCGACGGTCAACTTCATGACCAA R′ GTCGACTTGTGGTCTAGGGAAACCGTAAAAGC	63 64	158 bp	
Rib3	F′ CCGTCAACGGAACCTGCCTGACGGT R′ TTGAAGGTGGTCAGGTTGTAAGTCTGCGGCAT	64 64	94 bp	
Rib4	F′ CTAACTGTGCGGCAACGTACTTGCC R′ GGAGGTTGGTTCCCACTCACCTATG	67 61	168 bp	
REC	F′ CACGTGCCGAAATTGAAGGTGAAATGGGTG R′ CACCAGGAGTCGTTTCAGGATTACCAAACAT	63 62	110 bp	
TUF	F′ GGTCCGATGCCACAAACTCGTGAACACAT R′ CGGACAGAAGGTCACGAACTTCCATTTCAAC	63 63	130 bp	


### RNA Extraction, cDNA Synthesis, and RT-PCR

Selected Lactobacilli were grown in 10 ml of MRS, Riboflavin Assay Medium (devoid of riboflavin) (RAM), Skim milk and Whey based medium. The RNA was extracted after 6, 12, 18, and 24 h of incubation using TRIzol reagent followed by cell lysis by lysozyme (10 mg/100 ml) (Sigma, USA) (Ramiah et al., 2007). The quality of isolated RNA was checked (Sambrook and Russell, 2001). RNA was quantified and its purity of RNA was judged and used for reverse transcription. The cDNA was prepared with cDNA kits (RevertAid^TM^ First strand c-DNA synthesis kit, Fermentas, India), according to the manufacturer’s instructions. SYBR Green I Master mix (Roche) on 2 μL of diluted cDNA (1:1) using exon-spanning primers, 5 μL of SYBR green buffer 2X (Roche) and 2.5 pmol of each primer (**Table 2**) for a total volume reaction of 10 μL were used for qualitative PCR. The amplification was run in Lightcycler^®^ 480 II, and the results were analyzed using Lightcycler^®^ 480 II software release 1.50 SP3. The PCR conditions were as follows: initial denaturation at 95°C for 10 min, followed by 40 cycles of amplification at 63°C for 30 s and 72°C for 30 s. At the end of the each run a melting curve was achieved from 70 to 95°C and continuous fluorescence measurement was taken. A melting curve analysis was performed in order to verify the specificity of real-time PCR (RT-PCR) and finally, a cooling step to 4°C was achieved. Fluorescence was measured once every cycle after the extension step using filters for SYBR Green I (excitation at 465 nm and emission at 510 nm). To calculate the threshold cycle value, the normalized fluorescence data was converted to a log scale and threshold value was determined. The quantitative data of RT-PCR is generated on the basis of number of cycles required for optimal amplification generated fluorescence to reach a specific threshold of detection (the quantification cycle:Cq values) (Bustin et al., 2009). The comparative critical threshold (ΔΔCT) method was used to calculate the relative expression ratios in which the amount of target RNA is adjusted to a reference (internal target RNA) (Livak and Schmittgen, 2001). The Prism 7.00 was used to analyze the RT-PCR data sets.

ΔCT =CT⁢of⁢internal⁢control−CT⁢of⁢gene⁢of⁢interest.

ΔΔCT =ΔCT⁢of⁢sample−ΔCT⁢of⁢reference.

Relative⁢expression⁢level =2ΔΔCT.

### Isolation of Roseoflavin-Resistant Strains

The roseoflavin-resistant strains of KTLF1 was performed (according to Burgess et al., 2004, 2006) by exposing wild strain to increasing concentrations of roseoflavin (Santa Cruz Biotechnology, Santa Cruz, CA, USA) in RAM. Further experiments were carried out by using subsequent inoculum in 1 mL of RAM supplemented with roseoflavin. From the culture grown at the maximum range of roseoflavin, 15 separated colonies were randomly isolated after spreading onto MRS agar plates, and those stocks were stored at -80°C in CDM roseoflavin-free supplemented with 20% of glycerol (Russo et al., 2014).

### Principal Component Analysis

To discriminate the riboflavin producing isolates and riboflavin biosynthesis genes on the basis of mRNA expression levels in different media and at different time and principal component analysis (PCA) treatment, IBM SPSS Statistics 21.0 software program (IBM, Armonk, NY, USA) was used (ANOVA followed by a Tukey’s *post hoc* test). *P* < 0.05 was considered as statistically significant.

## Results

### Cloning Transformation and Sequencing

Purified amplicons were ligated into PTZ57R/T cloning vector. The ligates were transformed into competent cells of *E. coli* XL1 blue strain. The recombinant clones showed white (recombinant) colonies on LB agar plates supplemented with ampicillin (100 μg/ml). Ten randomly selected recombinant clones were analyzed by colony PCR for *Rib* genes. Consequently, positive clones were used for plasmid DNA analysis and isolated plasmids were further confirmed for their size by PCR and subsequently sequenced (Supplementary File). The sequences obtained from the isolates were compared by BLAST analysis for similarity check with three reference strains of *Lactobacillus* submitted to NCBI GenBank (Supplementary File). No size variation was found in the studied genes in different lactobacilli after staining the PAGE with silver staining (Supplementary File).

### Growth of Isolates in MRS, RAM, Milk and Whey for RNA Isolation and Gene Expression

**FIGURE 1:**
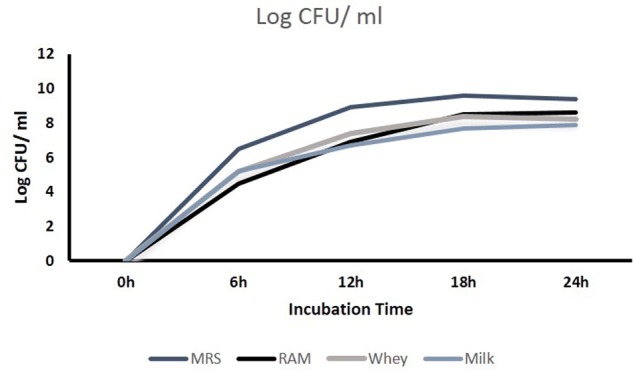
**Growth characteristics of KTLF1 in four different media**.

**FIGURE 2:**
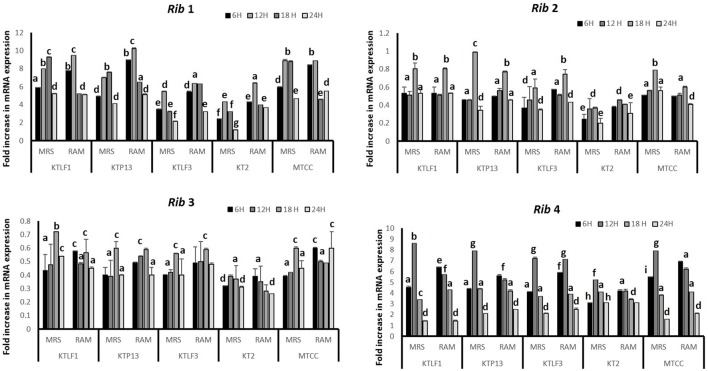
**Comparative mRNA expression profile (fold increase) of four test isolates with respect to reference culture (relative expression is considered as 1) in MRS and RAM.** Values are mean ± SD (*n* = 3) and different letters denote the significant difference at *P* < 0.05 (abcd refer to statistical differences with respect to MRS and RAM. Strain MTCC8711 is taken as control.

**FIGURE 3:**
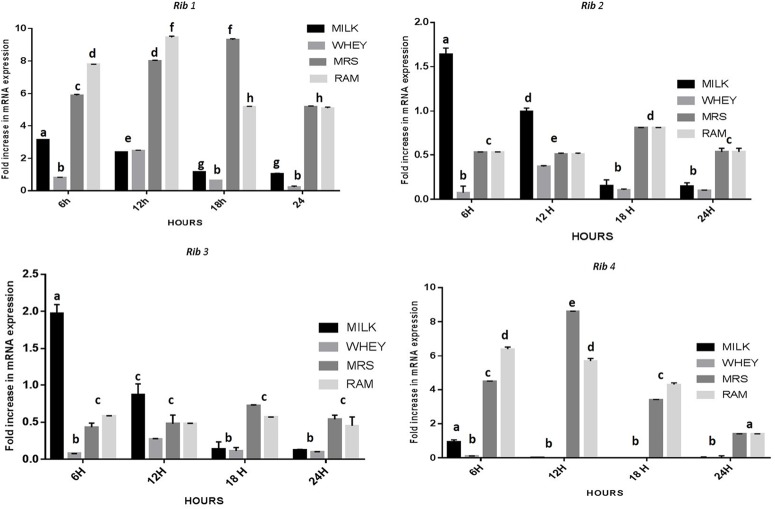
**Comparative mRNA expression profile (fold increase) of KTLF1 in MRS, RAM, Whey and Milk.** Values are mean ± SD (*n* = 3) and different letters denote show the significant difference at *P* < 0.05.

**FIGURE 4:**
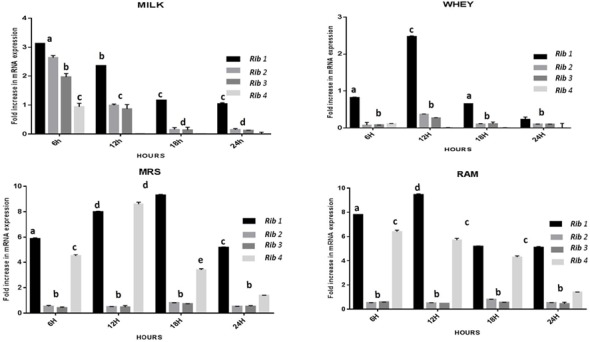
**Overall fold increase in relative mRNA expression of riboflavin structural genes in KTLF1 with respect to media and time intervals over control (MTCC8711).** Values are mean ± SD (*n* = 3) and different letters denote show the significant difference at *P* < 0.05.

**FIGURE 5:**
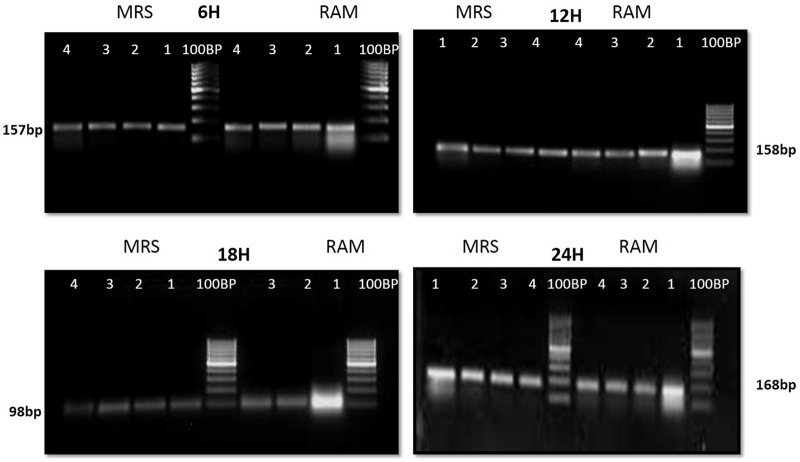
**1.5% agrose gel electrophoresis to discriminate the band intensity after RT-PCR in KTLF1 where 1, 2, 3, and 4 denote *rib* 1, *rib* 2, *rib* 3, and *rib* 4 genes**.

The CFU/ml was observed for KTLF1 at different growth phases (**Figure 1**) and the mRNA and the cDNA were prepared as described above. During the early growth, expression levels remain low and get elevated at later phase. The expression levels of *Rib* 1, *Rib* 4 were significantly higher in all the media used at four time intervals (**Figure 2**), whereas *Rib*2 and *Rib* 3 have shown almost constant regulation with different variables (Isolates, Media, and Time) (**Figure 2**). After incubation in different media at different time, the level of mRNAs expression was found to be changed in all the tested isolates. Particularly in KTLF1 strain, the mRNA expression level increased significantly in MRS at 12–18 h, in RAM at 6–12 h, in Milk at 6 h and in whey the upregulation was observed at 12 h (**Figure 3**). Among all the media used, RAM has shown increase in relative expression followed by MRS, Milk and Whey (**Figure 4**). Overall, there was no statistical difference in expression levels of *Rib*2 and *Rib*3 at different variables but a gradual decrease in the mean expression with time was recorded. **Figure 5** revealed the marked difference of increase in the intensity of RT-PCR products at different variables in KTLF1 in MRS and RAM which correspond significantly to change in mRNA expression profile. In order to study the effect of different parameters mentioned on riboflavin production, relative expression levels of the *Rib* 1, *Rib* 2, *Rib* 3, and *Rib* 4 genes were calculated in comparison with lowest riboflavin producing strain obtained from our previous study. The gene expression profiles for each strain grown under these conditions were different (**Figure 2**). Among the five tested strains, KT2 has shown significant variation in gene expression profile across the different incubation time intervals. The *Rib* 3 and *Rib*4 genes had a steady transcript level (fold change equal to one) in all the tested strains except in KT2 strain. The most significant difference was found at 6 and 24 h. This showed that expression of these genes was not much affected with the strain in two different media. Whereas, in KTLF1 (**Figure 3**), effect of media on different genes was significant in Milk and whey however, the relative expression levels of these genes was steady in MRS and RAM across different incubation. In particular, the expression levels of these genes were found to be lowered with increased incubation time in all the four media. In KTLF1, all the genes have shown significant variation in their expression in milk, Whey and MRS (**Figure 4**). In milk and whey, the fold increase was found in descending order with the increased incubation times, whereas, in whey media, except *Rib* 1, remaining three genes have shown steady expression over different incubation periods. In MRS and RAM, except *Rib* 1 and *Rib* 4, remaining two genes have shown significant change in expression profile.

### Principal Component Analysis

**FIGURE 6:**
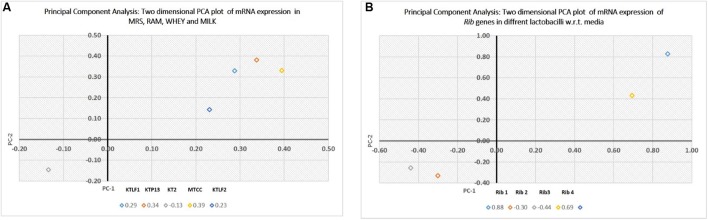
**(A)** Principal component analysis (PCA), expressed as two dimensional plot with respect to media as variables for the first two principal components. **(B)** PCA, expressed as two dimensional plot with respect to *Rib* genes for the first two principal components.

The multivariate analysis was used for comparison of experimental data obtained for five lactobacilli for mRNA expression of four *Rib* genes (PCA). Two dimensional plots are drawn in **Figure 6**. In plot 1 (**Figure 6A**), contribution of the media with respect to variance is shown. The first two components present the total variance of 53.7%. The discrimination of isolates along PC1 is mainly due to RAM and Whey. Whereas along PC2, the isolate KT2 is discriminated vis-à-vis other isolates. In plot 2 (**Figure 6B**), the first two components present the total variance of 73.7%. The discrimination along samples along PC1 is mainly due to *Rib* 1 and *Rib* 4, whereas *Rib* 2 and *Rib* 3 are responsible for discrimination along PC2.

### mRNA Expression of *Rib* Genes in Roseflavin Resistant Variant KTLF1 (4) and Riboflavin Overproduction

**FIGURE 7:**
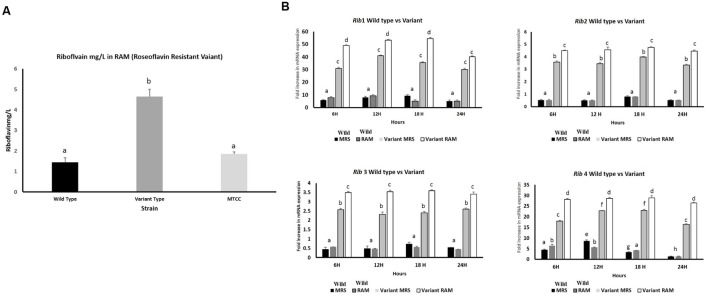
**(A)** Riboflavin production by roseoflavin-resistant KTLF (4) and wild strain in RAM. The line above the bar represents the SD of the mean and different letters denote show the significant difference at *P* < 0.05. **(B)** Over expression of *Rib* genes after exposure to roseoflavin in KTLF (4). Values are mean ± SD (*n* = 3) and different letters denote show the significant difference at *P* < 0.05.

By following the procedure described by Burgess et al. (2006), KTLF1 was exposed to roseoflavin, a structural analog of riboflavin, which induces mutations in riboflavin-producing strains leading to a novel producer phenotype of the vitamin. Roseoflavin resistant variants were isolated and inoculated in the riboflavin-free medium and incubated for 16 h at 30°C. Both wild type and variant strains were re-inoculated in MRS, RAM, Milk and Whey based media at 37°C, 24 h and harvested for RNA isolation and riboflavin production. From five isolated variants, only KTLF1 variant [KTLF1 (4)] was able to up regulate mRNA expression of *Rib* genes and the increase in riboflavin production was more than 3.5-fold (4.5 mg/L) as compared with wild type strain in a culture medium without riboflavin (**Figure 7A**). The spike in mRNA expression level was observed after the exposure of roseoflavin in all the four genes across the media at different times of incubation (**Figure 7B**). The maximum up regulation was observed in *Rib*1 followed by *Rib*4. The fold increase in expression was lower as to that of other wild type strains (**Figure 7B**).

## Discussion

Consumers are increasingly becoming conscious for their nutritional requirements, thus, vitamins produced *in situ* by microbes may suit their needs and expectations (LeBlanc et al., 2013; Thakur et al., 2015b). Since little information is available on what factors affect riboflavin production in LAB, the aim of this study was to investigate the influence of incubation time and difference strains on the expression of the rib genes by a wild type strains. Also, the vitamin production by the roseoflavin resistant strain under these conditions was also evaluated in mutant strain. As it is known that riboflavin operon is inducible, it is essential to evaluate the mRNA levels *rib* genes in different strains in different media (MRS, RAM, Milk and Whey) at different interval of growth time (6, 12, 18, and 24 h). The isolates used in this study are prospected from dairy, non- dairy sources (fermented bamboo shoots, human feces) (Thakur and Tomar, 2015a). The isolate KTLF3 was isolated from fermented bamboo shoots collected from Manipur, North East Region (Ethnic) of India. The riboflavin producing isolates used in this study are well-characterized by *in vitro* methods for their functional probiotic (Thakur and Tomar, 2015b) as well as technological properties (Thakur et al., 2016c). Arena et al. (2014) have also reported the probiotic lactobacilli for riboflavin production as well as its overproduction by using roseoflavin. In our study, riboflavin-producing strains were selected on the basis of mRNA expression of riboflavin biosynthesis genes. Out of four strains, three strains were able to show change in relative expression in the targeted genes with different incubation and media variables. Thus, KTLF1 was confirmed as prolific riboflavin producer and hence was selected to screen the mRNA profile of these genes in milk and whey based media. Milk and whey enriched in riboflavin have shown the increase in relative expression after the riboflavin in the media was utilized by the bacteria for its growth. The riboflavin may be required by bacteria in small amounts, but it constitutes a vital growth factor for *Enterococcus faecalis*, *Streptococcus pyogenes*, *Listeria monocytogenes*, and some lactobacilli (Koser, 1968). The biosynthetic deficiency correlates well with the absence of riboflavin in the growth media as the riboflavin operon is an inducible one where the quantity of riboflavin inhibits its production by bacteria. Unlike, other media, the RAM has shown higher expression levels which is in accordance with the aforementioned hypothesis.

KTLF1 was further selected to observe the overexpression of *Rib* genes after exposure to certain levels of roseoflavin. These results clearly indicated the roseoflavin exposure led to 3.5-fold increase in riboflavin production besides increase in the expression of all the rib genes by the mutant strain compared with those obtained with wild type strains, being more marked this difference at **Figures 7A,B**. The tolerance to the toxic roseoflavin signifies the mutations in the regulatory region of the rib operon which ultimately give rise to riboflavin overproducing phenotype. Till date, riboflavin overproduction is led either by employing genetic engineering (Burgess et al., 2004, 2006). The increase in expression levels of *Rib*1 and *Rib*4 followed by *Rib*2 and *Rib*3 is due to increased transcription of riboflavin biosynthesis genes because riboflavin metabolism and transport genes are being regulated at transcription attenuation. Further, threefold (4.5 mg/L) increase in riboflavin production in a culture medium without riboflavin was in agreement with the all these reports. Overexpression of all the four genes contributes to enhanced riboflavin production (Burgess et al., 2004) which was also observed in our study. Roseoflavin-resistant strains of *Leu. mesenteroides* over produced up to 0.5 mg l^-1^ of riboflavin, whereas riboflavin-overproducing *Lactobacillus plantarum* and *Propionibacterium freudenreichii* were able to synthesize up to around 0.6 and 3 mg l^-1^ respectively (Burgess et al., 2006). According to del Valle et al. (2014) roseoflavin resistant strains increased six times (1860 ± 20 ng/mL) the initial riboflavin levels of soy milk.

The PCA plots have well-discriminated the isolates on the basis of their levels of mRNA expression. The *Rib* genes are also put into two different groups due to their high and low expression with respect to variables. Overall, this study reveals that isolates showed variations for expressing their *Rib* genes which qualifies riboflavin production as strain specific attribute.

## Conclusion

The expression profile as well as phenotypic production of riboflavin have revealed that both the genotypic and phenotypic traits are dependent on riboflavin in media used for growth. Though the genotypic as well as phenotypic expression of riboflavin in milk and whey is lower as compared to riboflavin free media but it is better to use riboflavin producing bacteria than riboflavin consuming ones. From this study, it is clear that milk and whey can be used for development of riboflavin enriched fermented products. To the best of our knowledge, the present study reports for the first time the mRNA expression profile of riboflavin biosynthesis genes in lactobacilli. Furthermore, exposure of roseoflavin led to over expression of *Rib* genes in the variant of KTLF1 as compared to wild strains facilitates the enhanced riboflavin content in the final product. Lactobacilli isolated from fermented bamboo shoots have shown highest riboflavin producing properties as well as displayed probiotic and appreciable techno-functional properties which can be further explored to develop functional bamboo shoot foods. The riboflavin enriched products could be introduced by using these isolates as starters to prevent or treat riboflavin deficiencies which are still to be addressed.

## Author Contributions

KT is the first author and has carried out the research work as a part of her Ph.D. program. The data analysis and manuscript writing was done by KT. ST has corrected the manuscript and helped in experimental work. Z-JW has helped revising the manuscript.

## Conflict of Interest Statement

The authors declare that the research was conducted in the absence of any commercial or financial relationships that could be construed as a potential conflict of interest.
