# Supplementary material for: Comparative mRNA Expression Profiles of Riboflavin Biosynthesis Genes in Lactobacilli Isolated from Human Feces and Fermented Bamboo Shoots
Source: Front Microbiol. 2017 Mar 17;8:427. doi: 10.3389/fmicb.2017.00427 (PMC5356473; doi:10.3389/fmicb.2017.00427)
Supplement: Supplementary file 1 [file Presentation_1.PDF]

Cloning and transformation of *Rib* genes

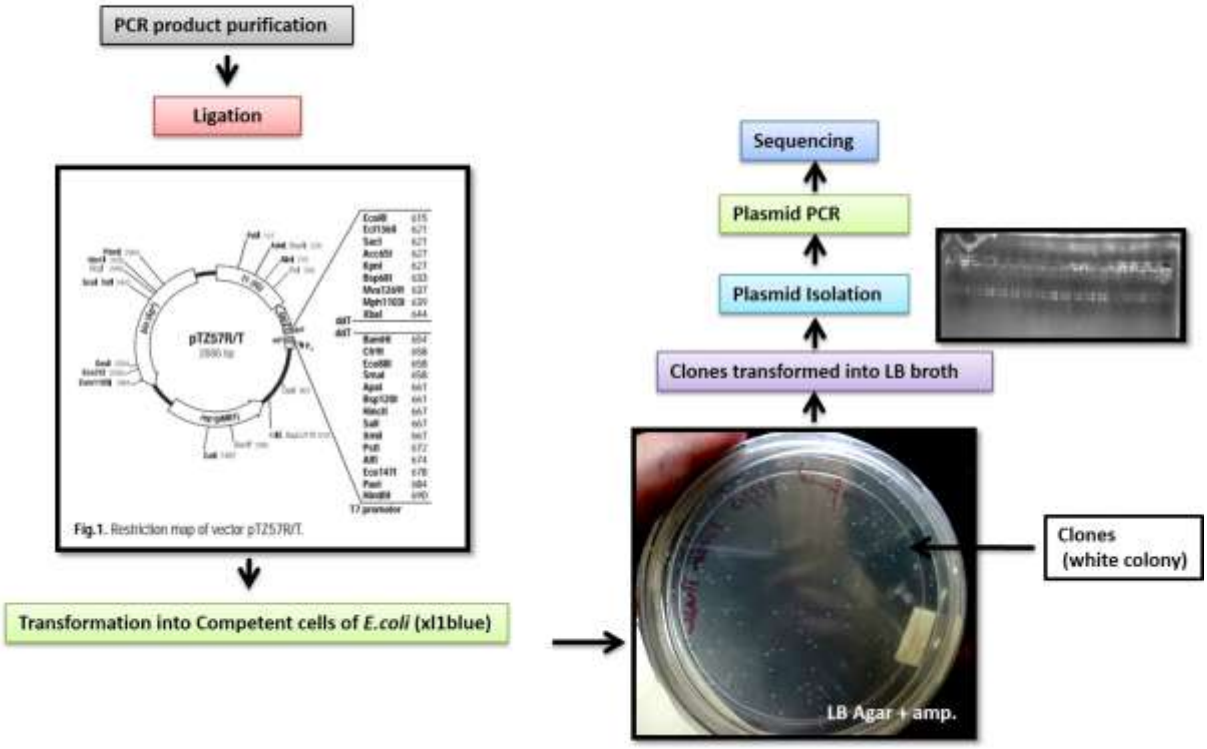

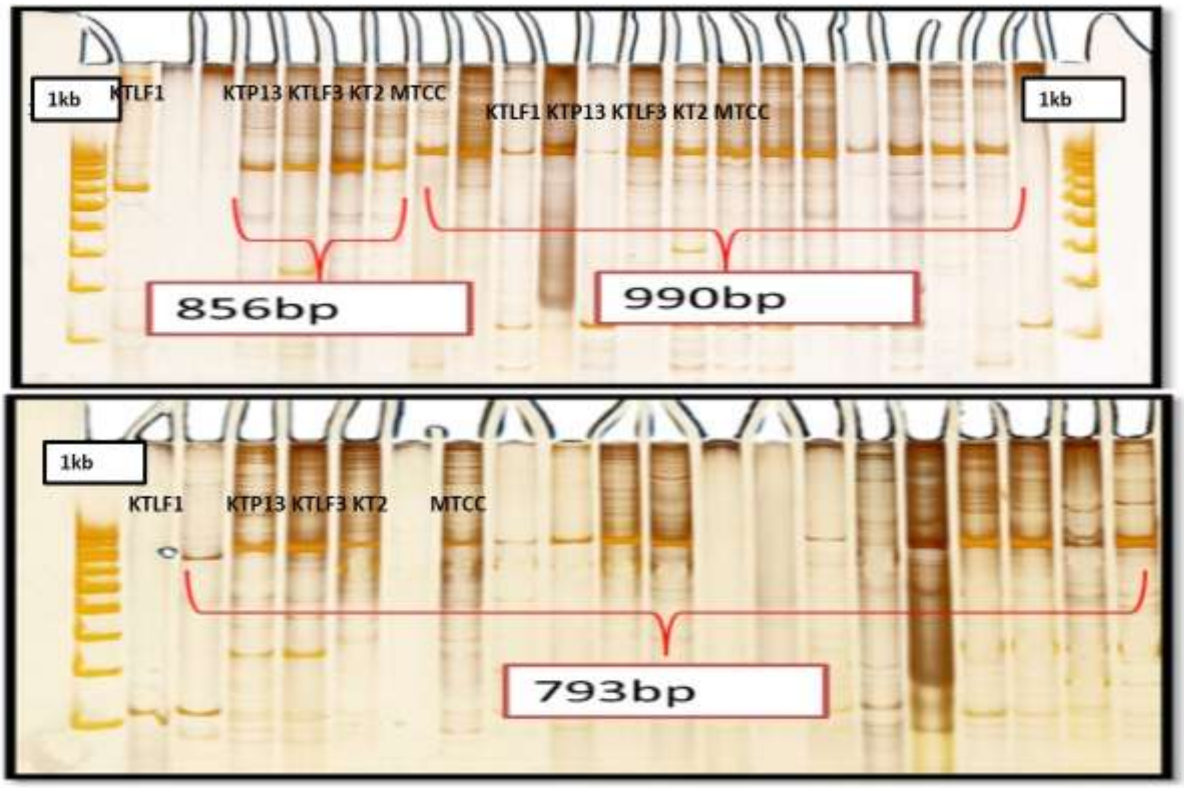


```
CLUSTAL 2.1 multiple sequence alignment Rib 1


IFO             CATCGAACCCTCGTTGCCGACCTTTAAGCCGGCCCGTTGCATCGCCTGTTCGATCGTGTC 60
5716            CATCGAACCCTCGTTGCCGACCTTTAAGCCGGCCCGTTGCATCGCCTGTTCGATCGTGTC 60
F-6             CATCGAACCCTCGTTGCCGACCTTTAAGCCGGCCCGTTGCATCGCCTGTTCGATCGTATC 60
KTF             ------------------------------------------------------------


IFO             GGTGGTCAAAATCCCGAAGGTCACCGGAATCGTGCCCTGAGCATTTAAGTTCATGATTGC 120
5716            GGTGGTCAAAATCCCGAAGGTCACCGGAATCGTGCCCTGAGCATTTAAGTTCATGATTGC 120
F-6             GGTGGTCAAAATCCCGAAGGTCACCGGAATCGTGCCCTGAGCATTTAAGTTCATGATTGC 120
KTF             ------------------------------------------------------------


IFO             GCTGGTGGTGGACTGGCAGATCAGGTCGTAGTGGTCGGTTTCGCCCTTGATCACGGCGCC 180
5716            GCTGGTGGTGGACTGGCAGATCAGGTCGTAGTGGTCGGTTTCGCCCTTGATCACGGCGCC 180
F-6             GCTGGTGGTGGACTGGCAGATCAGGTCGTAGTGGTCGGTTTCGCCCTTGATCACGGCGCC 180
KTF             ----------------------------------------TCGCCCTTGATCACGGCGCC 20
                                                        ********************

IFO             CAAGGTCATCACCCCGGCGTAGCGGCCGCTGGCGACCAGTTTCTTGGCGGCAAAGGCGAT 240
5716            CAAGGTCATCACCCCGGCGTAGCGGCCGCTGGCGACCAGTTTCTTGGCGGCAAAGGCGAT 240
F-6             CAAGGTCATCACCCCGGCGTAGCGGCCGCTGGCGACCAGTTTCTTGGCGGCAAAGGCGAT 240
KTF             CAAGGTCATCACCCCGGCGTAGCGGCCGCTGGCGACCAGTTTCTTGGCGGCAAAGGCGAT 80
                ************************************************************

IFO             TTCAAAGGCGCCTGGCACCCAGACGAGGTCAATTTGGTCGTCCTTGATCCCGAATTGCTT 300
5716            TTCAAAGGCGCCTGGCACCCAGACGAGGTCAATTTGGTCGTCCTTGATCCCGAATTGCTT 300
F-6             TTCAAAGGCGCCTGGCACCCAGACGAGGTCAATTTGGTCGTCCTTGATCCCGAATTGCTT 300
KTF             TTCAAAGGCGCCTGGCACCCAGACGAGGTCAATTTGGTCGTCCTTGATCCCGAATTGCTT 140
```

```
                   ************************************************************

IFO                TAGGGTCCGGATCGCCCCGTCGGCTAGGTTGCGGGTGACGGATTCGTTGAATTTACCAAC 360
5716               TAGGGTCCGGATCGCCCCGTCGGCTAGGTTGCGGGTGACGGATTCGTTGAATTTACCAAC 360
F-6                TAGGGTCCGGATCGCCCCGTCGGCTAGGTTGCGGGTGACGGATTCGTTGAATTTACCAAC 360
KTF                TAGGGTCCGGATCGCCCCGTCGGCTAGGTTGCGGGTGACGGATTCGTTGAATTTACCAAC 200
                   ************************************************************

IFO                GACGATCGCGATCTTCTTGGTGGGGGTGGTGGTAAAGTTTCCTTCGAATTGGTTAGGCAT 420
5716               GACGATCGCGATCTTCTTGGTGGGGGTGGTGATAAAGTTTCCTTCGAATTGGTTAGGCAT 420
F-6                GACGATCGCAATCTTCTTGGTGGGGGTGGTGGTAAAGTTTCCTTCGAATTGGTTAGGCAT 420
KTF                GACGATCGCGATCTTCTTGGTGGGGGTGGTGGTAAAGTTTCCTTCGAATTGGTTAGGCAT 260
                   ********* ********************* ****************************

IFO                GTTGTTCTTCCTCCGTGAGTTTGAGTAGGTGGTGCATCTTTTCTTGCTTGGTCTTGAGGT 480
5716               GTTGTTCTTCCTCCGTGAGTTTGAGTAGGTGGTGCATCTTTTCTTGCTTGGTCTTGAGGT 480
F-6                GTTGTTCTTCCTCCGTGAGTTTGAGTAGGTGGTGCATCTTTTCTTGCTTGGTCTTGAGGT 480
KTF                GTTGTTCTTCCTCCGTGAGTTTGAGTAGGTGGTGCATCTTTTCTTGCTTGGTCTTGAGGT 320
                   ************************************************************

IFO                AGGCGCGGTCGTAGGCGGTCGCCGGCATTTCCAGCGGCACCCGGGCTGCGACCGTGATCC 540
5716               AGGCGCGGTCGTAGGCGGTCGCCGGCATTTCCAGCGGCACCCGGGCTGCGACCGTGATCC 540
F-6                AGGCGCGGTCGTAGGCGGTCGCCGGCATTTCCAGCGGCACCCGGGCTGCGACCGTGATCC 540
KTF                AGGCGCGGTCGTAGGCGGTCGCGGGCATTTCGAGCGGCACCCGGGCTGCGACCGTGATCC 380
                   ********************** ******** ****************************

IFO                CGGCTTCTTCCAGTTGACTGATCTTATCGGGGTTGTTGGTCAGGAGTTTGATGGTTGAAA 600
5716               CGGCTTCTTCCAGTTGACTGATCTTATCGGGGTTGTTGGTCAGGAGTTTGATGGTTGAAA 600
F-6                CGGCTTCTTCCAGTTGACTGATCTTATCGGGGTTGTTGGTCAGGAGTTTGATGGTTGAAA 600
KTF                CGGCTTCTTCCAGTTGGCTGATCTTATCGGGGTTGTTGGTCAGGAGTTTGATGGTTGAAA 440
                   **************** *******************************************

IFO                CGTGGAGCGCCCTTAGGATGTGGCTAGCGGCCTCGTAGGTCCGTTCGTCGGCGGCAAAGC 660
5716               CGTGGAGCGCCCTTAGGATGTGGCTAGCGGCCTCGTAGGTCCGTTCGTCGGCGGCAAAGC 660
F-6                CGTGGAGCGCCCTTAGGATGTGGCTAGCGGCCTCGTAGGTCCGTTCGTCGGCGGCAAAGC 660
KTF                CGTGGAGCGCCCTTAGGATGTGGCTAGCGGCCTCGTAGGTCCGTTCGTCGGCGGCAAAGC 500
                   ************************************************************

IFO                CCAGTTGGTGGT 672
5716               CCAGTTGGTGGT 672
F-6                CCAGTTGGTGGT 672
KTF                CCAGTTGGTGGT 512
                   ************
```

Fig 1.1 Nucleotide sequence alignment of Rib 1 gene (KTLF1) with three reference sequences. The nucleotide changes are highlighted

```
CLUSTAL 2.1 multiple sequence alignment Rib 2


CECT            --------------------------------------------CGG-----GTCCCCCT 11
IFO             -------------------------------------------TCGG-----GTCCCCCT 12
CP005958.1      GTGAAGCACTCGGAGTGGACCCGGACCAACGGGGTAGTCGTGGTCGG-----GTCCCCCT 55
KTLF            ---------------------TGGACGTTCATAAGATTCAAG--CGGCCCTTACCCACCT 37
                                                            ***       ** ***

CECT            TGATCA---GGGCCAGGTTGCCGT------CGTCAAAG-TGGCGTAGGGTAAAGTCGCC- 60
IFO             TGATCA---GGGCCAGGTTGCCGT------CGTCAAAG-TGGCGTAGGGTAAAGTCGCC- 61
CP005958.1      TGATCA---GGGCCAGGTTGCCGT------CGTCAAAG-TGGCGTAGGGTAAAGTCGCC- 104
KTLF            TAAAAACGGGGGCCTGATCATCATGACCGACGACGAAGACCGCGAGGCAGAAGGTGACCT 97
                * *  *   ***** * *   * *      ** * ***   ***  *   ** **  **

CECT            ----GTACGCGCTGGGCAGGTGGACC-GGCGG-GACCGGTTGGCTGACCACCGAACGCCG 114
IFO             ----GTACGCGCTGGGCAGGTGGACC-GGCGG-GACCGGTTGGCTGACCACCGAACGCCG 115
CP005958.1      ----GTACGCGCTGGGCAGGTGGACC-GGCGG-GACCGGTTGGCTGACCACCGAACGCCG 158
KTLF            GTTAGGAATCGCCAGCCAGGTCACCCCGGCGACGGTCAACTTCATGACCAA-GCACGCCC 156
                    * *  ***  * *****   ** ****  *  *   *   ******  * *****

CECT            GTACTCTTGCAGTTCTTTGAC--GGTCAG------GATCGGCAACCGCAGTT---CCTCA 163
IFO             GTACTCTTGCAGTTCTTTGAC--GGTCAG------GATCGGCAACCGCAGTT---CCTCA 164
CP005958.1      GTACTCTTGCAGTTCTTTGAC--GGTCAG------GATCGGCAACCGCAGTT---CCTCA 207
KTLF            GCGGCCTCGAGCCTCTGTGCCCCGGTTAGCGAAGCGATCGCCAACCGCAACTGGGCCTGG 210
                *    **    * *** ** *  *** **      ***** *****  ***  *   ***

CECT            GCCATCTTCTCTAAGACCGGGCGCCGGGCCATGTGGCCGTCGGCGTCGAGAATTTCGCAG 223
IFO             GCCATCTTCTCTAAGACCGGGCGCCGGGCCATGTGGCCGTCGGCGTCGAGAATTTCGCAG 224
CP005958.1      GCCATCTTCTCTAAGACCGGGCGCCGGGCCATGTGGCCGTCGGCGTCGAGAATTTCGCAG 267
KTLF            ATTTAATGGTCCAAGACGCAACACCGAGCCCTACCACCACCGCTTTTACGGTTTCCCTAG 267
                      *  ** *****    * *** *** *    **  **   *   *  ** *  **

CECT            AT-----GTAG-CCGGCACCAGGCTCACCAGCCAACTCGGCCAGGTCGACGGCGGCTTCG 277
IFO             AT-----GTAG-CCGGCACCAGGCTCACCAGCCAACTCGGCCAGGTCGACGGCGGCTTCG 278
CP005958.1      AT-----GTAG-CCGGCACCAGGCTCACCAGCCAACTCGGCCAGGTCGACGGCGGCTTCG 321
```

```
KTLF            ACCACAAGTCGACCAGCACCGGAATC-TCGGCCTATTCGACCGGGCCGCCACCATCAAGG 324
                *      ** * ** ***** *  **  * *** * ** * ** ** ***  *   *  *

CECT            GTGTGGCCG---TTACGGGC---CAAGACGCCGTT--CT-CCTTGGCGATCAGCGGGAAG 328
IFO             GTGTGGCCG---TTACGGGC---CAAGACGCCGTT--CT-CCTTGGCGATCAGCGGGAAG 329
CP005958.1      GTGTGGCCG---TTACGGGC---CAAGACGCCGTT--CT-CCTTGGCGATCAGCGGGAAG 372
KTLF            CCCTGGCCGACCCCACCAGCACCCACGACGACTTTTACTACCCCGGCCA-CATCTTCCCG 383
                   ******     **  **   ** **** * **  ** **  *** * ** *     *

CECT            ATG-TGGCCGGGGTAGTAAAAGTCGTCGTGGGTGCTGGTGGGGTCGGCCAGGGCCTTGAT 387
IFO             ATG-TGGCCGGGGTAGTAAAAGTCGTCGTGGGTGCTGGTGGGGTCGGCCAGGGCCTTGAT 388
CP005958.1      ATG-TGGCCGGGGTAGTAAAAGTCGTCGTGGGTGCTGGTGGGGTCGGCCAGGGCCTTGAT 431
KTLF            CTGATCGCCAAGGCAGCGAACGGCGTCTTGGCTGCCCGTAAGGTCGGCCACACCGAAGCC 434
                 ** * ***  ** **  ** * **** *** **  *  *****    ******   * *

CECT            GGTGGCGGCCCGGTCA--TAGGCCGA-GATTCCGGTGCTGGTCGACTTGTGGTCTAGGGA 444
IFO             GGTGGCGGCCCGGTCA--TAGGCCGA-GATTCCGGTGCTGGTCGACTTGTGGTCTAGGGA 445
CP005958.1      GGTGGCGGCCCGGTCA--TAGGCCGA-GATTCCGGTGCTGGTCGACTTGTGGTCTAGGGA 488
KTLF            GCCGTCGACCTGGCCGAGTTGGCTGGTGAGCCTGGTGCCGGACTACTTGTGATCTGCGAA 488
                *  * ** ** ** *   * *** *  **  * ***** ** * ******* ***  ***

CECT            AACCGTAAAAGCGGTG-TGGTAGGGCTCGGTGTT--GTCTTGGACCATTAAATCCAGGCC 501
IFO             AACCGTAAAAGCGGTG-TGGTAGGGCTCGGTGTT--GTCTTGGACCATTAAATCCAGGCC 502
CP005958.1      AACCGTAAAAGCGGTG-TGGTAGGGCTCGGTGTT--GTCTTGGACCATTAAATCCAGGCC 545
KTLF            ATTCTCGACGCCGACGGCCACATGGCCCGGCGCCCGGTCTTAGAGAAGATGGCTGAGGCC 546
                *  *   *   **  *     * *** *** *    ***** **  *        *****

CECT            CAGCTGCTTGGCGATCGCTTCGCTAACCGGGGCACAGAGC----AGGCCGCGGGCGTGCT 557
IFO             CAGCTGCTTGGCGATCGCTTCGCTAACCGGGGCACAGAGC----AGGCCGCGGGCGTGCT 558
CP005958.1      CAGTTGCTTGGCGATCGCTTCGCTAACCGGGGCACAGAGC----AGGCCGCGGGCGTGCT 601
KTLF            -AACTGCTTGGTTACCGATCC--TGACCGT--CAAAGAACTGCAAGAGTACCGGCGTTCG 599
                 *  ***** **  * ** * *  * ****   ** *** *    **    * ***** *

CECT            -TGGTCATGAAGTTGACCGTCGCCGGGGTGACCTGGCTGGCGATTCCTAACAGGTCACCT 616
IFO             -TGGTCATGAAGTTGACCGTCGCCGGGGTGACCTGGCTGGCGATTCCTAACAGGTCACCT 617
CP005958.1      -TGGTCATGAAGTTGACCGTCGCCGGGGTGACCTGGCTGGCGATTCCTAACAGGTCACCT 660
KTLF            GTGGTCAGCCAACCGGTCGCCGCCGGT-CCACCTGCCCAGCGCGTACGGCGACTTTACCC 657
                 ******   *   *  **  ******    ***** *  ***  * *    *  * ***

CECT            TCTGCCTCGCGGTCTTCGTCGTC--GGTCATGAT-------GATCAGGCCCCCGTTTTTA 667
IFO             TCTGCCTCGCGGTCTTCGTCGTC--GGTCATGAT-------GATCAGGCCCCCGTTTTTA 668
CP005958.1      TCTGCCTCGCGGTCTTCGTCGTC--GGTCATGAT-------GATCAGGCCCCCGTTTTTA 711
KTLF            TACGCCACTTTGACGACGGCAACCTGGCCCTGATCAAGGGGGACCCGACCACGACTACCC 717
                *  *** *   * *  ** *  *  ** * ****       ** * * ** *   *

CECT            AGGTGGGTGAGGGCCGCTT-GAATCTTATGAACGTCCA 704
IFO             AGGTGGGTGAGGGCCGCTT-GAATCTTATGAACGTCCA 705
CP005958.1      AGGTGGGTAAGGGCCGCTT-GAATCTTATGAACGTCCA 748
KTLF            CGTTGGTCCGGGTCCACTCCGAGTGCTTCACA------ 749
                 * ***    ** ** **  ** *  *    *
```

Fig 1.2 Nucleotide sequence alignment of Rib 2 gene (KTLF1) with three reference sequences. The conserved nucleotides are presented by star*.

```
CLUSTAL 2.1 multiple sequence alignment Rib 3


KTF             ------------------------------------------------------------
CECT            CTAAGTTGATACAGGTTGGTAACGGTCTGGGTGTGGGGGATCAGGCCGACCGAGAACCAG 60
IFO             CTAAGTTGATACAGGTTGGTAACGGTCTGGGTGTGGGGGATCAGGCCGACCGAGAACCAG 60
F-6             ------------------------------------------------------------


KTF             ---------------------------CTGACCCCGTTGATCGCCACGCTCCCCTGGCTA 33
CECT            TCGTCTGCTTGCTTCATGACCGTCAAGCTGACCCCGTTGATCGCCACGCTCCCCTGGCTA 120
IFO             TCGTCTGCTTGCTTCATGACCGTCAAGCTGACCCCGTTGATCGCCACGCTCCCCTGGCTA 120
F-6             --------------------CGTCAAGCTGACCCCGTTGATCGCCACGCTCCCCTGGCTA 40
                                           *********************************

KTF             ATGACTTGCCCCCGCAACTCGTCAGGGAGCTTAAAGGTCAATTCAATTGCGTTTTCGTTA 93
CECT            ATGACTTGCCCCCGCAACTCGTCAGGGAGCTTAAAGGTCAATTCAATTGCGTTTTCGTTA 180
IFO             ATGACTTGCCCCCGCAACTCGTCAGGGAGCTTAAAGGTCAATTCAATTGCGTTTTCGTTA 180
F-6             ATGACTTGCCCCCGCAACTCTTCAGGGAGCTTAAAGGTCAATTCAATCGCGTTTTCGTTA 100
                ******************** ************************** ************

KTF             ACCTCCCGTTTGATGACCGGAGTCGTTTGGTCGACGTGCCT--CGACGATGTGGCCCTCC 151
```

```
CECT            ACCTCCCGTTTGATGACCGGAGTCGTTTGGTCGACGTGGCCGGTGACGATGTGGCCCTCC 240
IFO             ACCTCCCGTTTGATGACCGGAGTCGTTTGGTCGACGTGGCCGGTGACGATGTGGCCCTCC 240
F-6             ACCTCCCGTTTGATGACCGGAGCCGTTTGGTCGACGTGGCCGGTGACGATGTGGCCCTCC 160
                ********************** *************** *    ****************

KTF             AGCCGGTCGCCGATCTTTAGCGAGCGCTCCAAGTTGACCAGGGCACCAACTGGCAGGTCC 211
CECT            AGCCGGTCGCCGATCTTTAGCGAGCGCTCCAAGTTGACCAGGGCACCAACTGGCAGGTCC 300
IFO             AGCCGGTCGCCGATCTTTAGCGAGCGCTCCAAGTTGACCAGGGCACCAACTGGCAGGTCC 300
F-6             AGCCGGTCGCCGATCTTTAGCGAGCGCTCCAAGTTGACCAGGGCACCAACTGGCAGGTCC 220
                ************************************************************

KTF             TTGAAGGTGGTCAGGTTGTAAGTCTGCGGCATCAGGGTGACGGTCAGGCGGCCGGGTTGT 271
CECT            TTGAAGGTGGTCAGGTTGTAAGTCTGCGGCATCAGGGTGACGGTCAGGCGGCCGGGTTGT 360
IFO             TTGAAGGTGGTCAGGTTGTAAGTCTGCGGCATCAGGGTGACGGTCAGGCGGCCGGGTTGT 360
F-6             TTGAAGGTGGTCAGGTTGTAAGTCTGCGGCATCAGGGTGACGGTCAGGCGGCCGGGTTGT 280
                ************************************************************

KTF             TCGGTCTCGACCGTCAGGCAGGTTCCGTTGACGGCTAACGACGCCCCGACTTCCATTCCC 331
CECT            TCGGTCTCGACCGTCAGGCAGGTTCCGTTGACGGCTAACGACGCCCCGACTTCCATTCCC 420
IFO             TCGGTCTCGACCGTCAGGCAGGTTCCGTTGACGGCTAACGACGCCCCGACTTCCATTCCC 420
F-6             TCGGTCTCGACCGTCAGGCAGGTTCCGTTGACGGCTAACGACGCCCCAACTTCCATTCCC 340
                *********************************************** ************

KTF             TCTTGCAGGGCGGGGTCGAGGTCGATTGTTAGGCGGATGGTGTGGTCGGTTTGCTCAATC 391
CECT            TCTTGCAGGGCGGGGTCGAGGTCGATTGTTAGGCGGATGGTGTGGTCGGTTTGCTCAATC 480
IFO             TCTTGCAGGGCGGGGTCGAGGTCGATTGTTAGGCGGATGGTGTGGTCGGTTTGCTCAATC 480
F-6             TCTTGCAGGGCGGGGTCGAGGTCGATCGTTAGGCGGATGGTGTGGTCGGTTTGCTCAATC 400
                ************************** *********************************

KTF             TGGGTGAGCTTGCTTGT-CCGTTTACTAAGCCAGAGAACATT 432
CECT            TGGGTGAGCTTGCCCGTTCCGTTTACTAAGCCAGAGAACATT 522
IFO             TGGGTGAGCTTGCCCGTTCCGTTTACTAAGCCAGAGAACATT 522
F-6             TGGGTGAGCTTGCCCGTTCCGTTTACTAAGCCAGAGAACATT 442
                *************  ** ************************
```

Fig 1.3 Nucleotide sequence alignment of Rib 3 gene (KTLF1) with three reference sequences. The nucleotide changes are highlighted

```
CLUSTAL 2.1 multiple sequence alignment Rib 4


IFO             GAGTTGCGGCGTCTCGCACAGCAGGCGTGGGTCGTCGGCAAGCACGGTGCCGGCGCCGAC 60
F-6             ------------------------------------------------------------
CECT            GAGTTGCGGCGTCTCGCACAGCAGGCGTGGGTCGTCGGCAAGCACGGTGCCGGCGCCGAC 60
KTF             ------------------------------------------------------------


IFO             CACGATCGCGTGGTAGTCAGCGCGTTCCTGGTGGACCAGCCGGCGGGCGGCCTGGTTAGT 120
F-6             --------------------CGCGTTCCTGGTGGACCAGCCGGCGGGCGGCCTGGTTAGT 40
CECT            CACGATCGCGTGGTAGTCAGCGCGTTCCTGGTGGACCAGCCGGCGGGCGGCCTGGTTAGT 120
KTF             ---------------------------------------------------CTGGTTAGT 9
                                                                   *********

IFO             GATCGCCGTCGCTTGCCCCGGGGCGGCTGCAACCTTGCCATCCAAGGAGAGGGCCTGCTT 180
F-6             GATCGCCGTCGCTTGCCCCGGGGCGGCTGCAACCTTGCCATCCAAGGAGAGGGCCTGCTT 100
CECT            GATCGCCGTCGCTTGCCCCGGGGCGGCTGCAACCTTGCCATCCAAGGAGAGGGCCTGCTT 180
KTF             GATCGCCCGCGCTTGCCCCGGGGCGGCTGCAACCTTGCCATCCAAGGAGAGGGCCTGCTT 69
                *******  ***************************************************

IFO             GGCCGTGATCCAGGGCCGCTGCTGGCGGTAAAAGTAGAAGTAGTGGCGGTTGAGGGCCTC 240
F-6             GGCCGTGATCCAGGGCCGCTGCTGGCGGTAAAAGTAGAAGTAGTGGCGGTTGAGGGCCTC 160
CECT            GGCCGTGATCCAGGGCCGCTGCTGGCGGTAAAAGTAGAAGTAGTGGCGGTTGAGGGCCTC 240
KTF             GGCCGTGATCCAGGGCCGCTGCTGGCGGTAAAAGTATT-GCAGTGGCGGTTGAGGGCCTC 128
```

```
                ************************************   * *******************

IFO             GGCTTGGTCCTTTAAGAGCCCGACCTCCACATCAATTCCGGCCGCCTTTAGCTGGGCGAT 300
F-6             GGCTTGGTCCTTTAAGAGTCCGACTTCCACATCAATTCCGGCCGCCTTTAGCTGGGCGAT 220
CECT            GGCTTGGTCCTTTAAGAGCCCGACCTCCACATCAATTCCGGCCGCCTTTAGCTGGGCGAT 300
KTF             GGCTTGGTCCTTTAAGAGCCCGACCTCCACATCAATTCCGGCCGCCTTTAGCTGGGCGA- 187
                ****************** ***** ********************************** 

IFO             TCCCTTGCCACCAACGACTTGGTGGGGGTCAATCGTGGCGACGACCACCCGGGCAAAGTG 360
F-6             TCCCTTGCCACCAACGACTTGGTGGGGGTCAATCGTGGCGACGACCACCCGGGCAAAGTG 280
CECT            TCCCTTGCCACCAACGACTTGGTGGGGGTCAATCGTGGCGACGACCACCCGGGCAAAGTG 360
KTF             -CCCTTGCCACCAACGACTTGGTGGGGGTCAATCGTGGCGACGACCACCCGGGCAAAGTG 246
                 ***********************************************************

IFO             GCTGTCAATTAACAACTGGCTACACGGAGGCTGCTTGCCGTAGTGAAAGCAGGGCTCCAG 420
F-6             GCTGTCAATTAACAACTGGCTACACGGAGGCTGCTTGCCGTAGTGAAAGCAGGGCTCCAG 340
CECT            GCTGTCAATTAACAACTGGCTACACGGAGGCTGCTTGCCGTAGTGAAAGCAGGGCTCCAG 420
KTF             GCTGTCAATTAACAACTGGCTACACGGAGGCTGCTTGCCGTAGTGAAAGCAGGGCTC--- 303
                *********************************************************   

IFO             GGTGACGTAAATCGTGGCCCCGGCCAGCTGTTCGTTAGAAAGCTTACTGATGGCGTCGCG 480
F-6             GGTGACGTAAATCGTGGCCCCGGCCAGCTGTTCGTTAGAAAGCTTACTGATGGCGTCGCG 400
CECT            GGTGACGTAAATCGTGGCCCCGGCCAGCTGTTCGTTAGAAAGCTTACTGATGGCGTCGCG 480
KTF             -GTGACGTAAATCGTGGCCCCGGCCAGCTGTTCGTTAGAAAGCTTACTGATGGCGTCGCG 362
                 ***********************************************************

IFO             CTCGGCGTGGGCCTGACCATACTGGTGGTGGTAGCCCGTCGCCAGCAGCTGGCCGTCTTT 540
F-6             CTCGGCGTGGGCCTGACCATACTGGTGGTGGTAGCCCGTCGCCAGCAGCTGGCCGTCTTT 460
CECT            CTCGGCGTGGGCCTGACCATACTGGTGGTGGTAGCCCGTCGCCAGCAGCTGGCCGTCTTT 540
KTF             CTCGGCGTGGGCCTGACCATACTGGTGGTGGTAGCCCGTCGCCAGCAGCTGGCCGTCTTT 422
                ************************************************************

IFO             GACTATCACGGCGCCGACCTGGGGGTTCTTCCAGGTGGCGCTCCCGCCCTTGGCCGCTTC 600
F-6             GACTATCACGGCGCCGACCTGGGGGTTCTTCCAGGTGGCGCTCCCGCCCTTGGCCGCTTC 520
CECT            GACTATCACGGCGCCGACCTGGGGGTTCTTCCAGGTGGCGCTCCCGCCCTTGGCCGCTTC 600
KTF             GACTATCCGGGCGCCGACCTGGCGGTTCTTCCAGGTGGCGCTCCCGCCCTTGGCCGGTTC 482
                *******  ************* ********************************* ***

IFO             AGCGAGCGCCAGTTGCATGAACTGTTCGTCTGAACTCAAGGTAATGACTTCCTT 654
F-6             AGCGAGCGCCAGTTGCATGAACTGTTCGTCTGAACTCAAGGTAATGACTTCCTT 574
CECT            AGCGAGCGCCAGTTGCATGAACTGTTCGTCTGAACTCAAGGTAATGACTTCCTT 654
KTF             AGCGAGCGCCAGTTGCATGAACTGTTCGTCTGAACTCAAGGTAACGACTTCCTT 536
                ******************************************** *********
```

Fig 1.4 Nucleotide sequence alignment of Rib 4 gene (KTLF1) with three reference sequences. The nucleotide changes are highlighted
